# Spatial distribution estimation of malaria in northern China and its scenarios in 2020, 2030, 2040 and 2050

**DOI:** 10.1186/s12936-016-1395-2

**Published:** 2016-07-07

**Authors:** Yongze Song, Yong Ge, Jinfeng Wang, Zhoupeng Ren, Yilan Liao, Junhuan Peng

**Affiliations:** School of Land Science and Technology, China University of Geosciences, Beijing, China; State Key Laboratory of Resources and Environmental Information System, Institute of Geographical Sciences and Natural Resources Research, Chinese Academy of Sciences, Beijing, China; Key Laboratory of Surveillance and Early Warning on Infectious Diseases, Chinese Center for Diseases Control and Prevention, Beijing, China; University of Chinese Academy of Sciences, Beijing, China

**Keywords:** Malaria, Genetic programming, Remote-sensing data, Future distribution prediction, Climate change scenarios, Optimization algorithm

## Abstract

**Background:**

Malaria is one of the most severe parasitic diseases in the world. Spatial distribution estimation of malaria and its future scenarios are important issues for malaria control and elimination. Furthermore, sophisticated nonlinear relationships for prediction between malaria incidence and potential variables have not been well constructed in previous research. This study aims to estimate these nonlinear relationships and predict future malaria scenarios in northern China.

**Methods:**

Nonlinear relationships between malaria incidence and predictor variables were constructed using a genetic programming (GP) method, to predict the spatial distributions of malaria under climate change scenarios. For this, the examples of monthly average malaria incidence were used in each county of northern China from 2004 to 2010. Among the five variables at county level, precipitation rate and temperature are used for projections, while elevation, water density index, and gross domestic product are held at their present-day values.

**Results:**

Average malaria incidence was 0.107 ‰ per annum in northern China, with incidence characteristics in significant spatial clustering. A GP-based model fit the relationships with average relative error (ARE) = 8.127 % for training data (R^2^ = 0.825) and 17.102 % for test data (R^2^ = 0.532). The fitness of GP results are significantly improved compared with those by generalized additive models (GAM) and linear regressions. With the future precipitation rate and temperature conditions in Special Report on Emission Scenarios (SRES) family B1, A1B and A2 scenarios, spatial distributions and changes in malaria incidences in 2020, 2030, 2040 and 2050 were predicted and mapped.

**Conclusions:**

The GP method increases the precision of predicting the spatial distribution of malaria incidence. With the assumption of varied precipitation rate and temperature, and other variables controlled, the relationships between incidence and the varied variables appear sophisticated nonlinearity and spatially differentiation. Using the future fluctuated precipitation and the increased temperature, median malaria incidence in 2020, 2030, 2040 and 2050 would significantly increase that it might increase 19 to 29 % in 2020, but currently China is in the malaria elimination phase, indicating that the effective strategies and actions had been taken. While the mean incidences will not increase even reduce due to the incidence reduction in high-risk regions but the simultaneous expansion of the high-risk areas.

## Background

With a massive population at risk and widely threatened areas, malaria is a serious parasitic disease worldwide. In developing nations of tropical and subtropical area, malaria has become one of the largest obstacles to socioeconomic advancement [1]. Approximately 3.2 billion people (43.8 % of the world’s population) living in more than 100 countries are threatened by malaria to varying degrees [2]. Great achievements have been made in fighting and eliminating malaria over the past few decades, for instance, insecticide-treated nets are the most widespread intervention and are responsible for malaria reduction in many endemic countries [3]. Malaria risk areas, however, have not varied significantly compared with those a half century ago. Research has indicated that 1.13 and 1.44 billion people globally are at risk for unstable and stable *Plasmodium falciparum* malaria, respectively [4], and 2.5 billion people worldwide are at risk for *Plasmodium vivax* malaria [5].

Climate change and corresponding environmental alterations have significantly influenced the variation and transmission of malaria [6–10]. With a reliably predicted future malaria scenario, the malaria incidence at different locations could be depicted, especially in high-risk and new outbreak areas, to propose malaria elimination strategies and develop health policies [11]. Global and regional studies on malaria prediction have shown that the effects of climate change on malaria vary spatially [9]. Thus, improving understanding of both the temporal and spatial dynamic effects of climate on malaria transmission is of great importance for reducing the disease burden and risks to human health [12, 13].

Predictor variables include various environmental and socioeconomic variables that contribute to the appearance and transmission of malaria, such as precipitation, temperature, elevation, water density index (WDI), and gross domestic product (GDP) [14–19]. Remote sensing techniques and products can be used to predict malaria incidence because the propagation processes of malaria, namely, the source of infection, route of transmission, and susceptible individuals, are affected by atmospheric and environmental conditions [16, 20–22]. These conditions not only affect the growth of parasites inside malaria vectors, but also directly affect the habitat conditions and transmission activities of the *Anopheles* vector [23–25]. Remote sensing has advantages over real-time monitoring of these conditions, such as its features of timeliness, wide monitoring range, and easier data acquisition compared with ground monitoring stations [21, 26–28]. Precipitation and land surface temperature (LST), combined with epidemiological data, are commonly used to model and predict conditions of malaria prevalence [20, 23, 29–33]. These alternative, remotely-sensed ecological indicators could directly reflect the relationships between malaria transmission and atmospheric and environmental variables [21, 25]. Research conducted in Kenya, Africa, has successfully predicted local seasonal malaria prevalence and transmission intensity [20]. In addition, research in the Horn of Africa and Eastern Africa suggests that the prediction accuracy of *P. falciparum* malaria transmission intensity reaches 75 % using these techniques [17]. With respect to predicting distribution and quantity of the *Anopheles* vector, remote sensing techniques could be used to determine mosquito breeding sites and predict malaria risk distribution, to assist in malaria control efforts [29, 34].

Precipitation and temperature, especially remotely-sensed precipitation rate (PR) and LST data, are particularly effective predictor variables because they have significant relationships with malaria incidence and their temporal delayed effects [35, 36]; they both are also important products of future climate change scenarios. Research in Huang-Huai River in China demonstrated the malaria re-emergence was significantly related to the change of local precipitation [37, 38]. A study in Guangdong Province, China, compared the median temperature with 30 °C and showed that temperature has an important role in malaria incidence with delayed effects lasting for 4 weeks (maximum relative risk (RR) of 1.57, 95 % confidence interval (CI) 1.06–2.33) [39]. Moreover, temperature has significant localized effects on malaria transmission [40], and the relationship between temperature and malaria incidence is affected by the various environmental conditions in a certain area [41].

While the majority of current research between malaria and environmental/socioeconomic variables focuses on linear modelling [24, 42], a few studies have revealed a nonlinear relationship in certain settings [18, 23]. Exploring the significance of malaria at various intervals using remote sensing data is a common experimental goal [22, 43, 44]. Therefore, exploring nonlinear relationships between malaria and predictor variables using nonlinear methods is important. Genetic programming (GP) is an optimization method that explores the ability to construct complex nonlinear relationships between certain problems and express them mathematically. GP is therefore effective in addressing sophisticated nonlinear issues, eliminating nonfunctional variables, and modelling a proper function structure closest to the truth [45, 46].

Northern China (Henan and Anhui provinces) is a typical mid-latitude, high-risk area of locally prevalent *P. vivax* malaria, which presents a great threat to the population of 170 million (2010). The number of malaria cases in China has decreased since 1950. The reported average malaria incidence had decreased to 0.194 ‰ *per annum* (p.a.) by the year 2000, and the number of cases has decreased to 24,088 in 2000 from over 24 million cases by 1970. These numbers began to rebound in 2000, exceeding 64,000 cases in 2006 when the incidence reached 0.50 ‰ p.a. Malaria in China has a distinct regional distribution, with northern China one of the areas with the highest prevalence [47]. In the present study, the influence of explanatory variables on malaria incidence in northern China and its future spatial distributions under climate change scenarios were predicted using the GP method, accompanied by geographic information system (GIS) methods for advanced spatial analysis and expression [48]. Predictor variables were remote sensing data of PR and LST, together with elevation, WDI, and GDP. In addition, China would achieve malaria elimination by 2020, Asia–Pacific region would achieve malaria-free by 2030 [2], and perhaps by 2050, as an ambitious goal, human malaria was expected to be eventually eliminated [49]. While if no strategies were implemented, population exposed to the primary malaria vectors would continuously increase in 2030s and 2050s in China [10]. Therefore, 2020, 2030, 2040 and 2050 were the years for projections owning to their significance regionally and globally. Assuming precipitation and temperature were the changed variable and other variables remained unchanged, future spatial distributions of malaria in the years 2020, 2030, 2040 and 2050 were predicted and mapped with the future temperature conditions in Special Report on Emission Scenarios (SRES) family B1, A1B and A2 scenarios [50].

## Methods

### Study area and malaria data

**Fig. 1:**
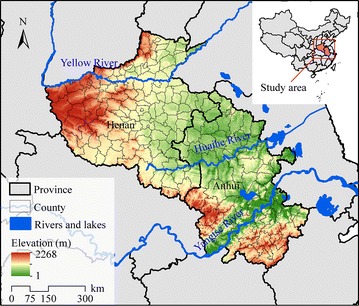
Study area consists of 205 counties in three provinces (Henan and Anhui) in northern China

The study area consisted of 205 counties in two provinces (Henan and Anhui) of northern China (Fig. 1). This area is located within 110.35–119.64°E and 29.40–36.37°N. About 170 million people live within 306,000 km^2^, in one of the most densely populated areas globally. Plains make up the predominant terrain in this area, with a few mountains located in western Henan Province and some hilly regions in Anhui Province. Three main rivers flow through this area, the Yellow, Huaihe, and Yangtze rivers.

The Chinese Center for Disease Control and Prevention (Chinese CDC) has summarized monthly malaria cases in each county from 2004 to 2010. In two provinces (Henan and Anhui) of northern China, the total number of malaria cases was 127,448, and average malaria incidence was 0.107 ‰ p.a. The total cases in Henan and Anhui provinces during the seven-year period were 19,182 and 108,266, respectively. With populations of 103 million in Henan and 66 million in Anhui, the annual average malaria incidence is 0.026 and 0.232 ‰ p.a., respectively. Areas with malaria incidence greater than 0.1 ‰ p.a. are considered stable risk areas, whereas those with incidence lower than 0.1 ‰ p.a. are considered areas of unstable risk [51, 52]. Among the seven-year average incidences in each county, the highest incidence reached 2.191 ‰ p.a. and the incidences in 22 counties were larger than 0.1 ‰ p.a. The study area was, therefore, a malaria high-risk area during these 7 years.

### Predictor variables and climate change scenarios data

With the advantages of remote sensing over real-time data, such as its wide spatial coverage and relatively easy acquisition, remotely sensed monitoring data have been applied to various malaria prediction problems [16, 21, 23, 53]. The remote sensing monitoring indexes in this research include monthly PR (mm/h) from the Tropical Rainfall Measuring Mission (TRMM) 3B43 (version 7) product with the spatial resolution of 0.25° (~25 km) [54], and monthly LST (°C) from the terra moderate resolution imaging spectroradiometer (MODIS) product MOD11A2 with the spatial resolution of 1 km downloaded from the level 1 and atmosphere archive and distribution system (LAADS Web) at NASA website [55]. Monthly PR are resampled and calculated to the data with the unit of mm/day and the spatial resolution of 5 km. Both remote sensing products are pre-processed to county level, meaning that the spatial average values of each county are calculated such that these variables reflect the average atmospheric or environmental conditions at county level. In addition, remotely sensed precipitation and temperature match the monthly malaria incidence data in time, from 2004 to 2010. Given that the occurrence and spread of malaria are not only influenced by meteorological and environmental variables, two kinds of easily obtained auxiliary data are used in this study, including geographical variables (elevation and WDI) and a social variable (GDP). Elevation data is derived from topographic dataset of Shuttle Radar Topography Mission (SRTM3) with a 90 m spatial resolution [56]. The percentage of the total area of rivers and lakes in a county is calculated as WDI, which is calculated with raster data from global land water regions dataset at 30 m spatial resolution [57]. GDP is sourced from 1 km Grid GDP Dataset of China (2010) [58]. They are all transformed to county level, corresponding to malaria data.

When averaging the variables over the counties, the impact of the variable variation within each county on the county-level transforming process is analysed by F-test. For instance, elevation varies within county *k* (*k* = 1, 2,…, 205), and its variation is depicted by 205 randomly selected spatial points of elevation values, which is the data *A*_*k*_. F-test is used to test the difference between *A*_*k*_ and the county-level averaged elevation data *B*. As such, the percentage of counties with significant difference comparing data *B* is used to depict the impact of the variable variation within each county on the averaging. Table 1 summarizes the percentages for elevation, GDP, PR, and LST with the significant p value of 0.01, 0.05 and 0.1, where PR and LST are the monthly data in 2004, and the performances of both monthly variables in other years are similar. It shows that the variations of variables in most counties are significantly different from those of the county-level averaged ones.

**Table 1:** Percentage of counties with significant difference between the varied data within each county and the averaged one

Variable	p value of significant difference			Variable	p value of significant difference		
	0.01 (%)	0.05 (%)	0.1 (%)		0.01 (%)	0.05 (%)	0.1 (%)
Elevation	86.34	88.78	90.24	GDP	59.51	66.34	70.73
PR in Jan	100.00	100.00	100.00	LST in Jan	95.12	95.61	96.10
PR in Feb	100.00	100.00	100.00	LST in Feb	93.66	95.12	95.61
PR in Mar	100.00	100.00	100.00	LST in Mar	76.10	81.95	87.32
PR in Apr	100.00	100.00	100.00	LST in Apr	76.59	82.44	85.37
PR in May	100.00	100.00	100.00	LST in May	83.90	88.29	89.76
PR in Jun	100.00	100.00	100.00	LST in Jun	96.10	96.59	97.07
PR in Jul	100.00	100.00	100.00	LST in Jul	89.27	92.68	94.63
PR in Aug	98.54	98.54	98.54	LST in Aug	90.73	92.20	93.17
PR in Sep	100.00	100.00	100.00	LST in Sep	89.76	93.17	94.63
PR in Oct	99.51	99.51	99.51	LST in Oct	95.12	96.59	97.07
PR in Nov	100.00	100.00 %	100.00	LST in Nov	100.00	100.00	100.00
PR in Dec	100.00	100.00 %	100.00	LST in Dec	100.00	100.00	100.00

The future precipitation and temperature are projected to change significantly under the SRES family B1, A1B and A2 scenarios. Future precipitation and temperature data of climate change scenarios were analysed by the a series of global climate models (GCMs), which were available by the CGIAR Research Program on Climate Change, Agriculture and Food Security (CCAFS) [59] based on the World Climate Research Programme’s (WCRP’s) Coupled Model Intercomparison Project Phase 3 (CMIP3) multi-model dataset [50, 60]. B1, A1B and A2 emission scenarios were included in CMIP3 for climate projection, each of which corresponds to a specific pathway to reach each target radiative forcing caused by long-lived and short-lived greenhouse gases [61]. The projected global average surface temperature changes at 2090–2099 relative to 1980–1999 are estimated to be 1.8, 2.8 and 3.4 °C under B1, A1B and A2 scenarios, respectively [62]. The future scenarios data with the spatial resolution of 5 km are also summarized to the county level in the study area.

### GP-based malaria incidence prediction

**Fig. 2:**
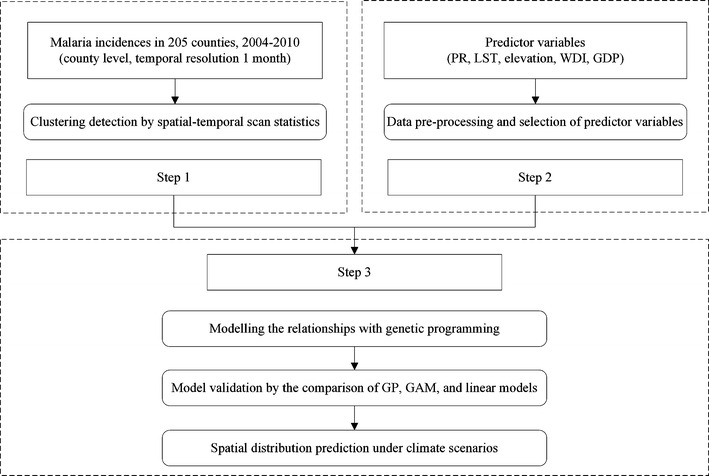
Schematic overview of GP-based malaria incidence prediction

The objective of GP-based prediction was to predict the spatial distributions of malaria incidence in northern China under climate change scenarios in the years 2020, 2030, 2040 and 2050, assuming that the PR and temperature variables were the varied ones among all variables for prediction. County was used as the spatial mapping unit corresponding to malaria case data; thus, both predictor variables and climate change scenario data were averaged for each county. The main steps of GP-based malaria incidence prediction are outlined in the schematic overview (Fig. 2). This process consisted of three steps: (1) spatiotemporal analysis for malaria incidence; (2) data pre-processing and selection of predictor variables; and (3) modelling and prediction with the GP method.

In the first step, spatiotemporal scan analysis was performed to identify and quantify the spatiotemporal clustering scales of malaria incidence in the study area [63, 64]. The basic theory of scan analysis used in epidemiology is as follows: building a moving scan window in space, calculating the total number of cases *C* and number of expected cases *E* both inside and outside a certain window, and estimating the difference between incidences in and out of the window through assessing log likelihood ratio *r* with the formula $r = ( {{c /{E(c)}}} )^{c} \times \{ {{{ ( {C - c} )} /{( {C - E(c)} )}}} \}^{{C -c}} \times I()$, where *c* is the number of actual cases and *I*() is an indicator function. During the scan process, when the number of cases is larger than the expected value, *I*() is 1; otherwise, it is 0. By dynamically changing the size and location of the window and recalculating *r* when new cases appear until a maximum *r* is selected, the window at this time is the clustering window of high incidence. The window size is depicted with the ratio of population within the window to the total population, which ranges from 0 to the maximum risk population that is set based on research and is less than 50 % of the total population. The result of spatiotemporal scan statistics is the accurate high-risk areas of prevalence.

The second step before GP prediction was to pre-process data and select reliable predictor variables. As a small probability event, malaria incidence summarized in spatial cross sections was 0 in many of the spatial units. The focus of spatial analysis is spatial cross-section data as well as the differences in various regions. Therefore, to select the proper variables, Spearman correlation coefficients (given rank information) between dependence and independence were calculated [65]. The effect of a 0 value was thus reduced; the information provided by 0 incidence was used fully and loss of information was decreased. Then, the multicollinearities of these explanatory variables were analyzed and as a result, variables with strong collinearities were removed [66, 67]. The variables and malaria incidence were significantly correlated, and the correlation coefficients of remote sensing indexes with 1-month lag effect reached maximum values (Table 2). After testing for normality and data transformation, the pre-processed predictor variables were X1 (RP, lag = 1), X2 (LST, lag = 1), X3 (log-transformed elevation), X4 (log-transformed WDI), and X5 (log-transformed GDP), which are statistically summarized in Table 3.

**Table 2:** Spearman correlations between 7-year average monthly malaria incidences and corresponding variables with lag effect

Lag	RP	LST	Elevation^a^	WDI^a^	GDP^a^	N
0	0.186**	0.207**	−0.330**	0.257**	−0.278**	2460
1	0.238**	0.235**	/	/	/	2460
2	0.212**	0.201**	/	/	/	2460

** Significant correlation (0.01)

^a^Transformed data

**Table 3:** Variables in the GP experiment

Variables	Minimum	Mean	Median	Maximum
X1	0.076	2.725	2.184	10.565
X2	−1.999	15.266	16.681	29.320
X3	2.216	4.539	4.257	7.011
X4	0.000	0.828	0.777	1.818
X5	−0.935	0.612	0.531	2.177

**Fig. 3:**
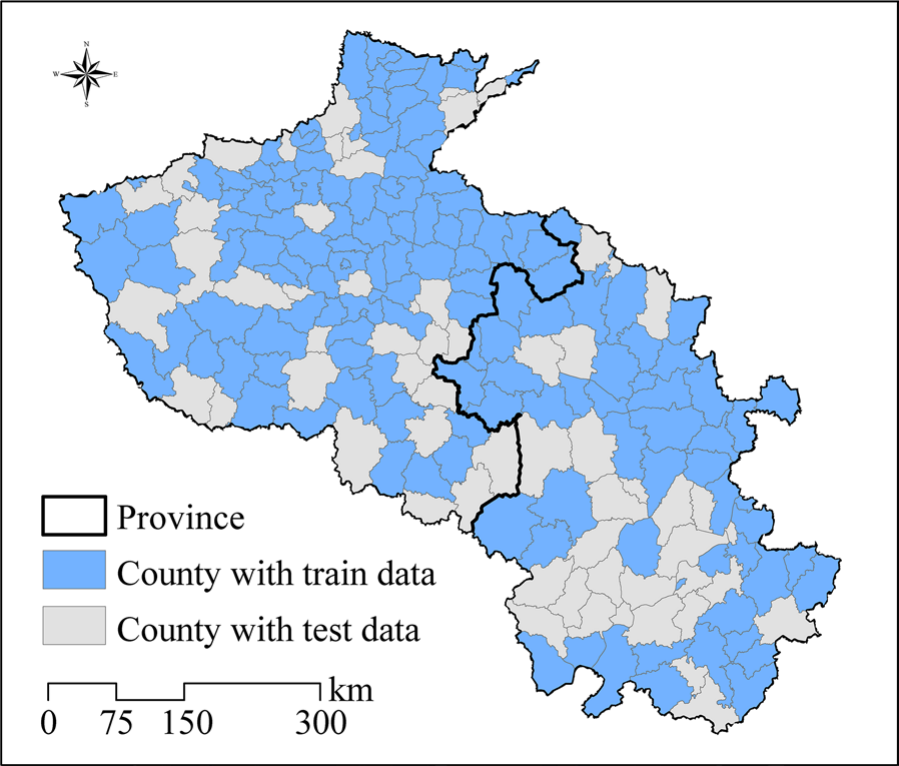
The distribution of randomly selected train counties and test counties

Finally, the relationships between malaria incidence and the corresponding predictor variables were constructed using the GP method. Based on this nonlinear relationship, the spatial distributions in 2020, 2030, 2040 and 2050 could be predicted and mapped under climate change scenarios. To ensure reliability, data in 70 % of the counties with monthly cases (144 counties) were randomly selected as training data, and data of the remaining 30 % (61 counties) were regarded as test data (Fig. 3). The fitted results would be affected by the quality of the parameter settings. General parameter settings for the GP framework are listed in Table 4. Terminal variables were X1, X2, X3, X4, and X5 and the function set was (+, −, × , /, power, log, exp, sqrt). At the beginning of the GP process, 200 equations were randomly generated with the terminal variables and the functions in the set to capture the relationship between malaria incidence and the predictors. These equations were the individuals in the initial population, and one of them with the best fitness would be selected. During GP process, because a better equation structure in the result required a lower fitness. The fitness function used in this experiment was the sum of the absolute difference (SAD), $fitness = \sum\nolimits_{i = 1}^{N} { | {y_{i} - {p_{i} } |} },$ where *N* was the total number of observations, and *y*_*i*_ and *p*_*i*_ were observed values and GP-predicted values, respectively. With the calculation of fitness, “winner” individuals were probabilistically transformed with crossover and mutation for parts of equations, to replace the “loser” ones, so that the individuals would be renewed in each next generation. The above steps were repeated until a program was developed that could reasonably predict malaria incidence [46, 48, 68]. GP was performed with 1000 generations in each monthly malaria incidence prediction case. At the end of the experiment, uncertainties were analysed to detect the precision and reliability of the research method. GPLAB, a genetic programming toolbox written for MATLAB software, was adopted to generate the prediction solution of malaria incidence [69]. A series of engineering and scientific problems have previously been addressed successfully using the GPLAB toolbox [68, 70–72]. To validate the performance of GP-based malaria incidence prediction model, the results of both generalized additive models (GAM) and linear regression are used for comparison. GAM is a common nonlinear model describing the nonlinear relationships via nonparametric smoothing functions [73], and it is performed by the *mgcv* package in the program R. For GAM and linear regression, the same predictors are used including the one-month lagged precipitation and temperature.

**Table 4:** General parameter settings for the GP experiment

Parameters	Descriptions and values
Terminal set	Variables X1, X2, X3, X4, and X5
Function set	+, −, × ,/, power, log, exp, sqrt
Population size	200 individuals
Generations	1000
Fitness function type	Sum of absolute difference (SAD)
Genetic operators	Crossover, mutation
Initialization	[0.85, 0.15]
Operators probability	Variable
Tree depth	Dynamic depth selection
Dynamic max depth	15
Real max tree depth	17
Selection method	Lexictour
Survival	Totalelitism (elistism)

## Results

**Fig. 4:**
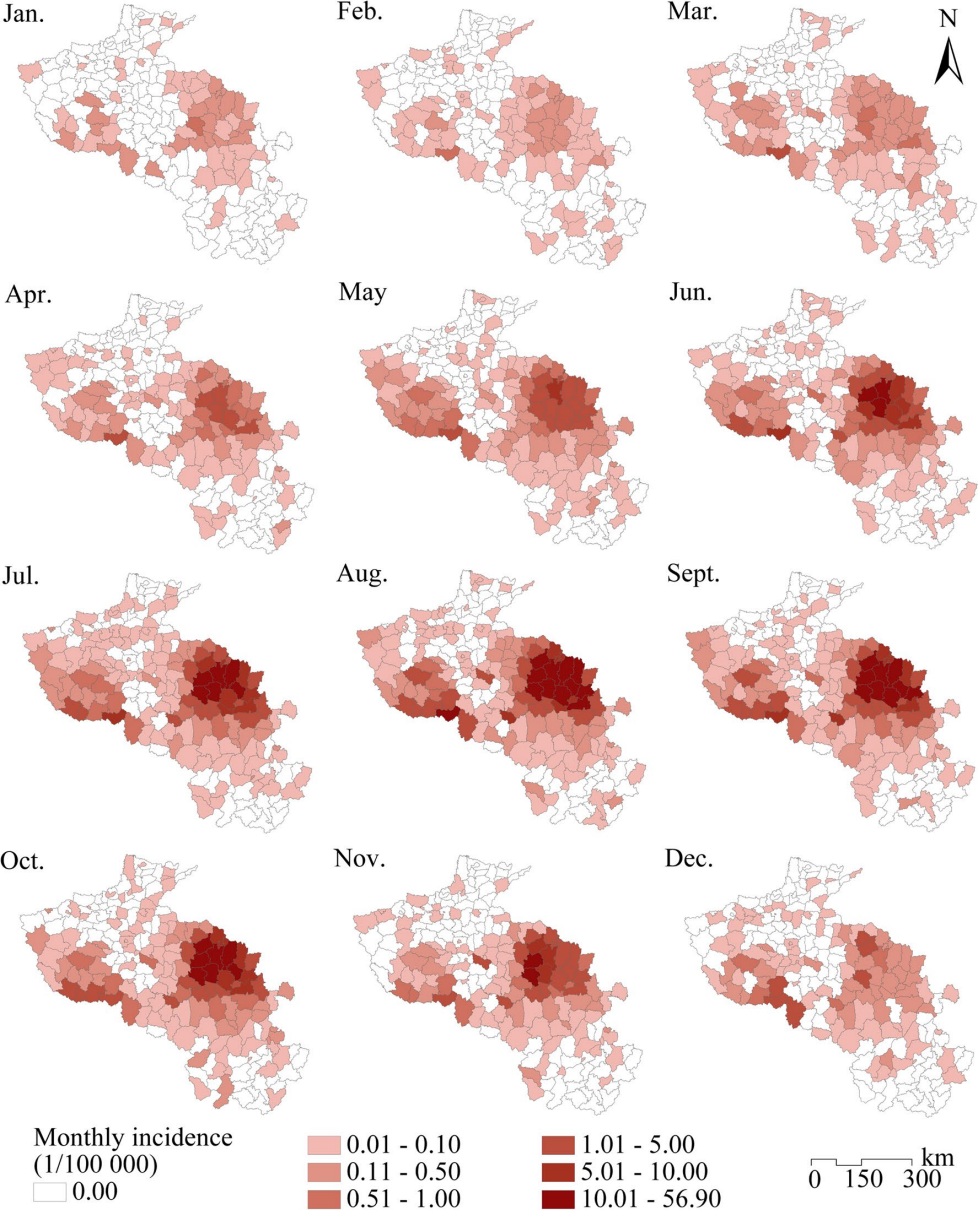
Monthly average malaria incidences of the statistics from 2004 to 2010

**Fig. 5:**
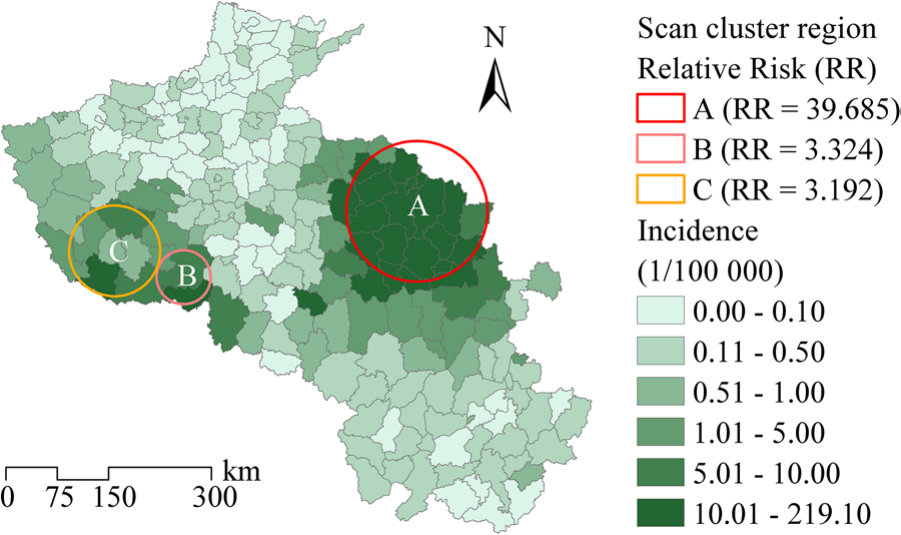
Three cluster regions detected by a spatiotemporal scan statistics method

Monthly average malaria incidence data were collected in 205 counties from 2004 to 2010, as shown in Fig. 4. This figure illustrates the spatial clustering and seasonality of malaria incidence in each county. The cluster regions detected by spatiotemporal scan statistics are mapped in Fig. 5; region A (16 counties) was the level one cluster region (radius 102.94 km), and regions B (2 counties) and C (7 counties) were the level two cluster regions (radius 39.42 and 66.30 km, respectively). In region A, located in northern Anhui Province, the cluster period was June 2005 to November 2008, the total number of cases was 88,242 (69.41 % of the total number in the study area), the RR was 39.69, and the log likelihood ratio was 181,306.01. The average malaria incidence was 1.366 ‰ pa, which was much larger than that in the entire study area from 2004 to 2010 (0.107 ‰ pa), and larger than the criterion of a stable malaria risk region (0.1 ‰ pa) [74]. Cluster regions B (cluster time frame, June 2004 to October 2007; total number of cases, 1682; incidence, 0.350 ‰ pa; RR, 3.32; log likelihood ratio, 838.75) and C (cluster time frame, August to September 2007; total number of cases, 442; incidence, 0.339 ‰ pa; RR, 3.19; log likelihood ratio, 209.13) were located in southwest Henan Province. The results showed that the cluster time frames of these three regions were in different months during different years, so it was necessary to consider the problem with monthly malaria incidence and the corresponding variables.

**Fig. 6:**
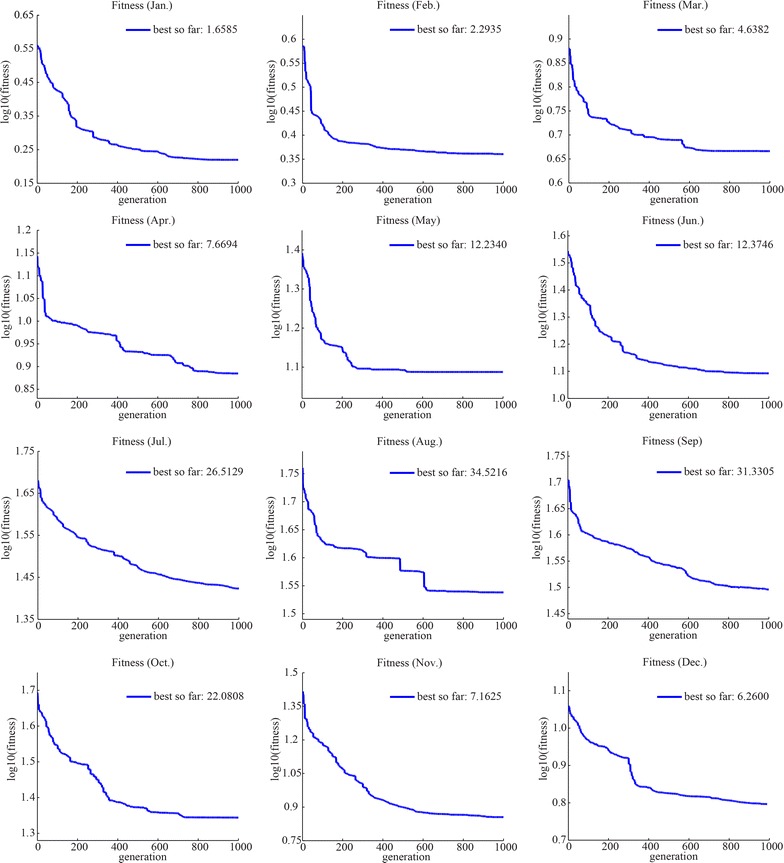
Fitness plots of the monthly average cases

**Fig. 7:**
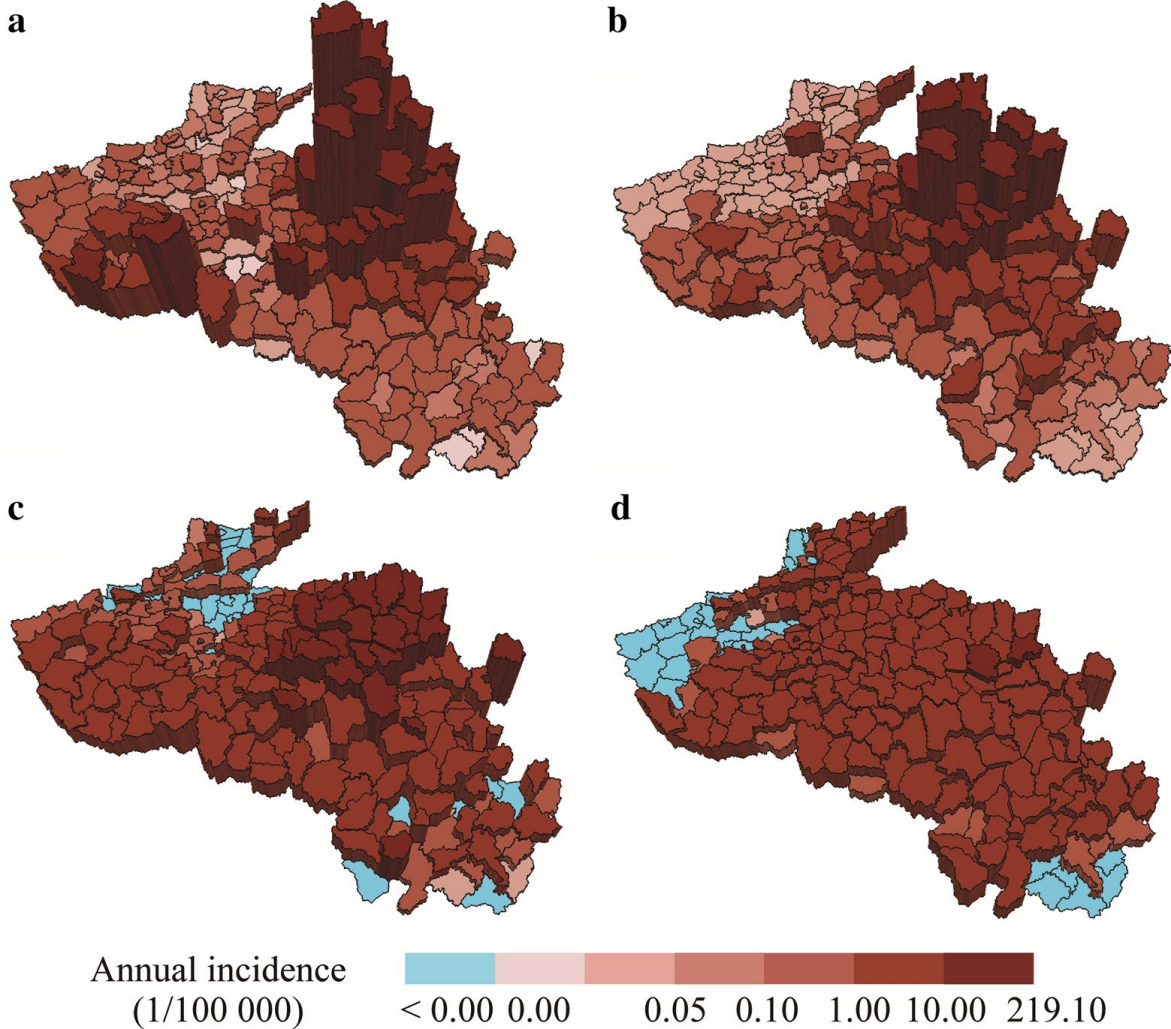
Comparison of maps of original and fitted annual average malaria incidence. Distributions of original incidences (**a**), GP fitted incidences (**b**), GAM fitted incidences (**c**) and linear regression fitted incidences (**d**)

A nonlinear relationship between county incidences and the five variables was constructed for the training data of each monthly case. Figure 6 presents the fitness of the best GP equation with the tree form composed by predictors and functions for each monthly case during the evolution process of 1000 generations. The relationships were then applied, to predict malaria incidence in the test counties with the five known variables. To compare the spatial distributions of GP-fitted malaria incidences in the training and test counties, these were summarized to annual average fitted values, mapped, and compared with the original dependent variable, the transformed incidence data. Figure 7 illustrates a map of the original transformed malaria incidence data (A), GP-fitted data (B), GAM-fitted data (C) and linear regression-fitted data (D). The patterns and trends of spatial distribution were rationally predicted with the GP method but were not predicted by GAM and linear regression.

**Fig. 8:**
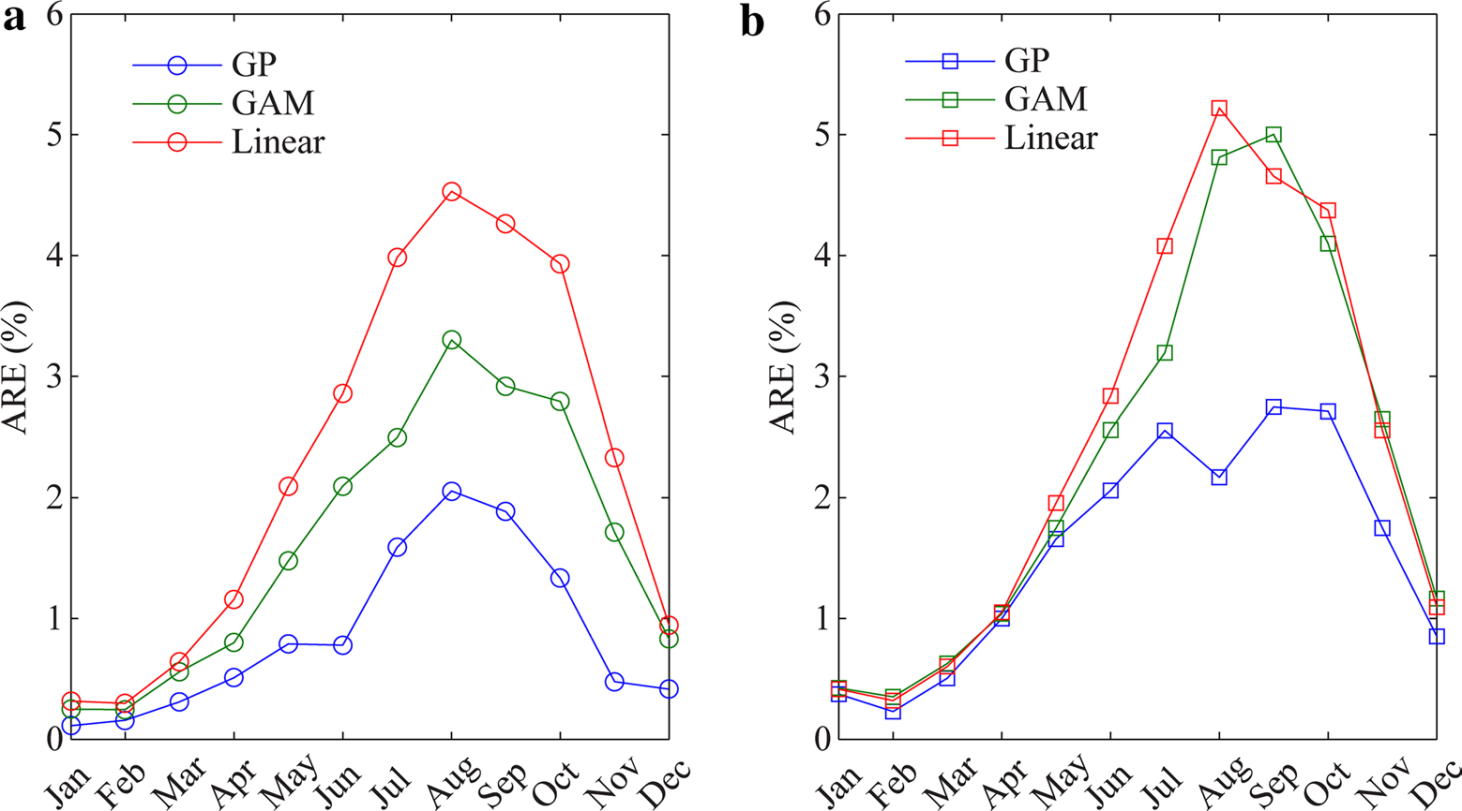
The ARE values of monthly fitting models for train data (**a**) and test data (**b**)

Table 5 presents prediction errors of the GP-based model, GAM-based model and linear regression approach for the monthly cases and the annual average cases. In the table, the average relative error (ARE) and mean sum squared error (MSSE) are defined by $ARE = \frac{1}{N}\mathop \sum \nolimits_{i = 1}^{N} \left| {\frac{{O_{i} - P_{i} }}{{(O_{i} + 10)}}} \right| \times 100$ and $MSSE = \frac{1}{N}\mathop \sum \nolimits_{i = 1}^{N} \left( {O_{i} - P_{i} } \right)^{2}$, where *O*_*i*_ and *P*_*i*_ denote observation and prediction for *i*th data, respectively; *N* is the total number of data items in the dataset. R^2^ describes the goodness-of-fit of the model which means the degree of association between the observed and model-simulated data. Among the three indexes evaluating prediction accuracy, ARE is more reliable owing to its focus on relative errors. Thus the ARE values of the monthly models are also validated as shown in Fig. 8. From the results evaluation table, it can be concluded that the GP method could more accurately predict malaria incidence, with ARE = 8.127 % for training data (R^2^ = 0.825) and ARE = 17.102 % for test data (R^2^ = 0.532), compared with GAM method ARE = 19.163 % for training data (R^2^ = 0.445) and ARE = 30.155 % for test data (R^2^ = 0.452), and linear regression ARE = 27.449 % for training data (R^2^ = 0.159) and ARE = 31.031 % for test data (R^2^ = 0.189). The ARE results in Fig. 8 also demonstrate that the fitness are significantly improved by the monthly GP model.

**Table 5:** Evaluation the results of GP, GAM, and linear regression models for the annual average case

Model	Train result			Test result		
	ARE (%)	MSSE	R^2^	ARE (%)	MSSE	R^2^
GP	8.127	264.180	0.825	17.102	311.229	0.532
GAM	19.163	540.100	0.445	30.155	392.238	0.452
Linear	27.449	690.995	0.159	31.031	490.594	0.189

**Fig. 9:**
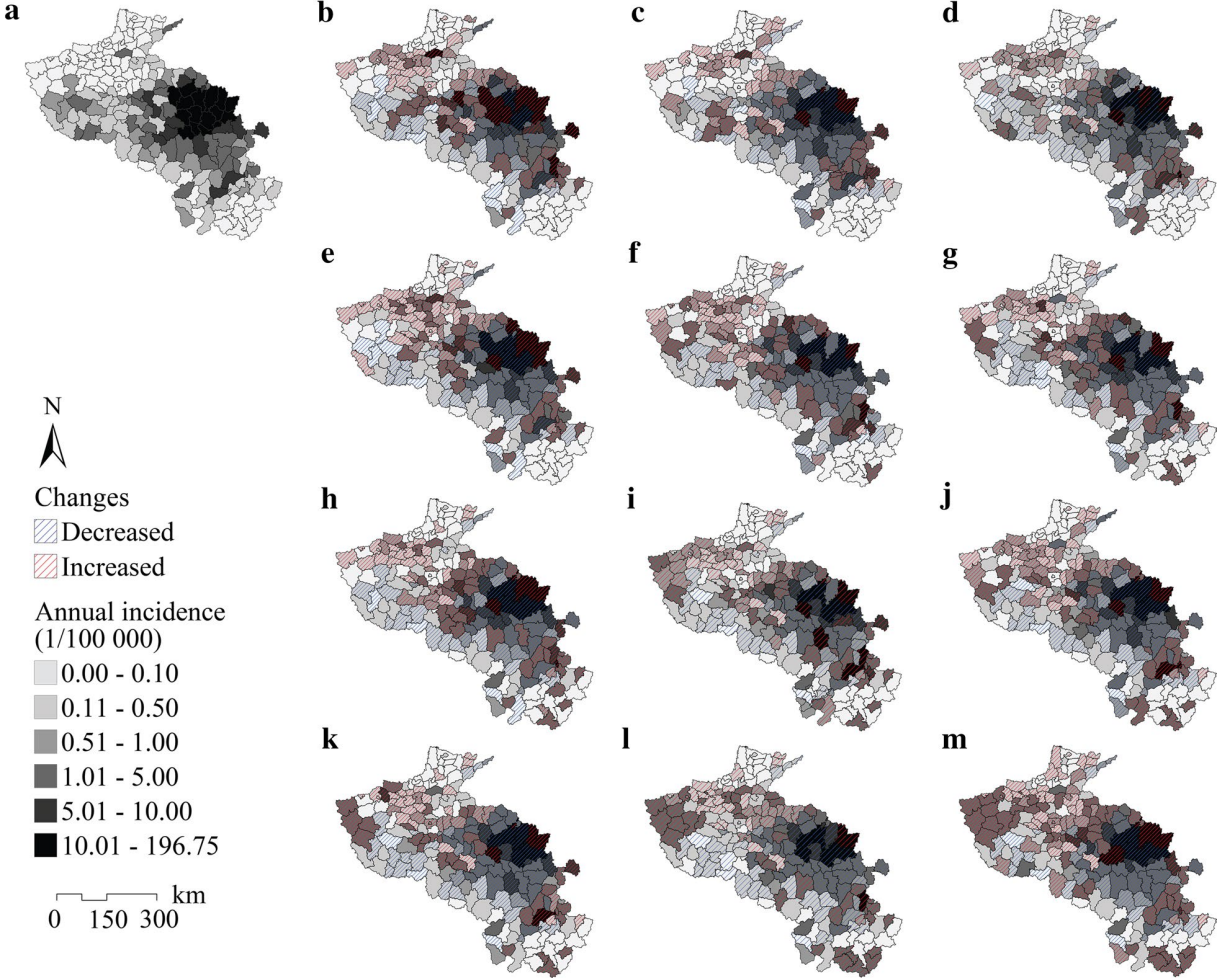
Malaria change maps under climate change scenarios. Distribution of average malaria incidence in 2004–2010 (**a**), distributions of incidence in 2020 (**b**, **c**, **d**), 2030 (**e**, **f**, **g**), 2040 (**h**, **i**, **j**) and 2050 (**k**, **l**, **m**) under B1, A1B, and A2 scenarios respectively

With the future PR and temperature conditions in the SRES family B1, A1B and A2 scenarios, the spatial distributions of malaria in 2020, 2030, 2040 and 2050 were predicted in each month of these four future years using a GP-based prediction model. Malaria incidence change maps are shown in Fig. 9, which depict changes in the predicted malaria incidence, especially the phenomenon of spatially increasing and decreasing incidence. Incidence changes were defined as the absolute values of changes greater than 0.001 ‰ p.a. (0.1/100,000).

## Discussion

**Fig. 10:**
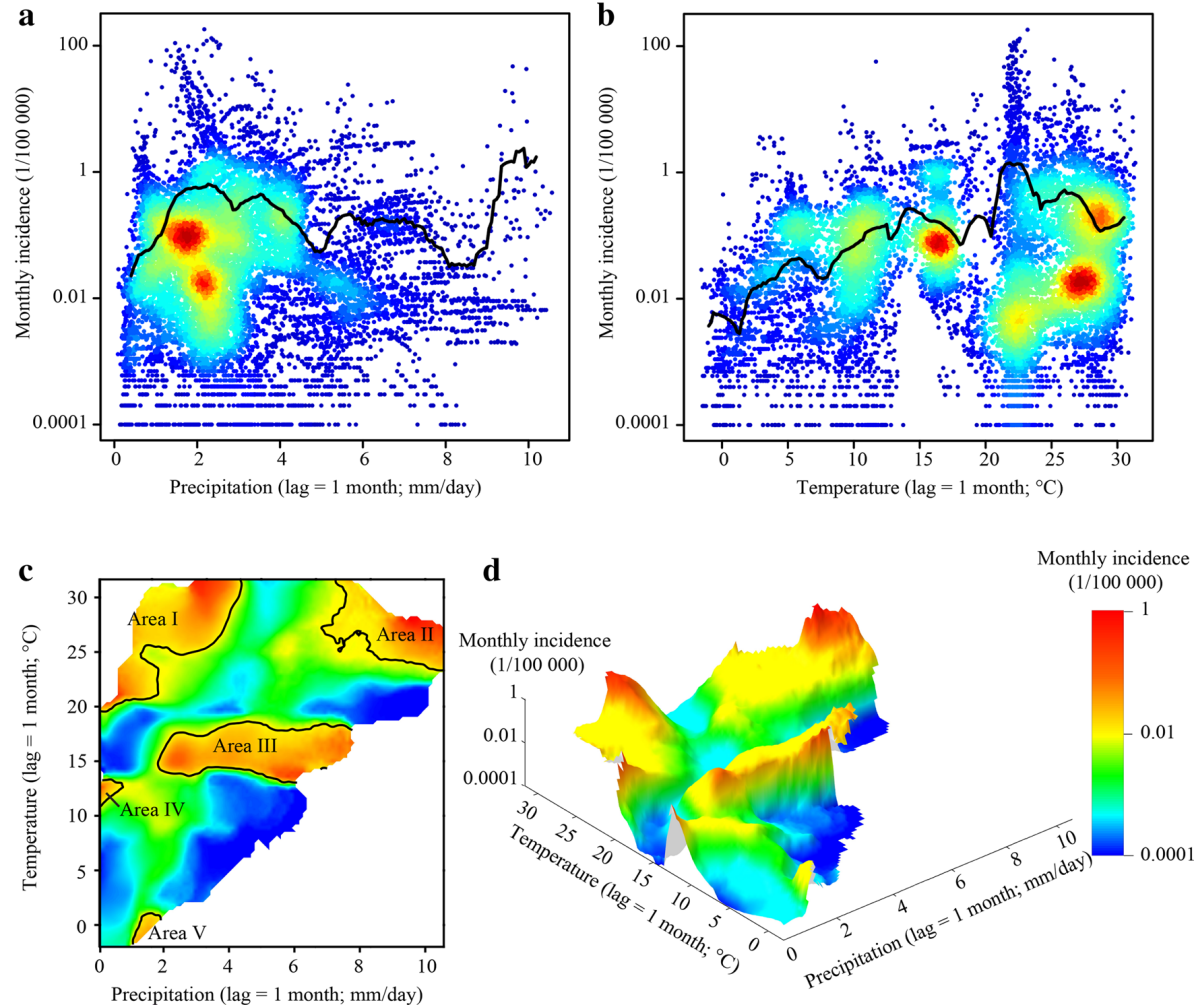
GP-predicted relationships between incidence and varied variables, precipitation and temperature, in northern China. Relationship between incidence and lagged precipitation (**a**), relationship between incidence and temperature (**b**), relationship between incidence and both variables (**c**), and its three-dimension expression (**d**)

The GP-based nonlinear model used in this study predicted the spatial distributions and changes of malaria incidence for the years 2020, 2030, 2040 and 2050 under SRES family B1, A1B and A2 climate scenarios with the assumption of a varied variables of precipitation and temperature, and the constant variables of elevation, WDI, and GDP. In the study area, to depict fluctuations in the nonlinear relationship between malaria incidence and varied precipitation and temperature with the other three variables remaining constant, monthly-predicted incidences were summarized to annual ones. The annual average incidence in each county was calculated with the precipitation and temperature set from the minimum to the maximum in the climate change scenarios. The relationships in all counties of the study area were then summarized. Figure 10 illustrates the summary by running mean monthly malaria incidences for each 0.07 mm/day of precipitation (Fig. 10a) and 0.19 °C of temperature (Fig. 10b), in which the incidences predicted by the GP-based model fluctuated with the increased lagged precipitation and temperature. There are four main peak values in the fluctuating relationship between incidence and precipitation with the precipitation of 2.2, 3.0, 5.7 and 9.8 mm/day, and a peak value in the nonlinear relationship between incidence and temperature with the temperature of 21.9 °C. In general, five primary high-incidence areas of thresholds with the running mean monthly incidence larger than 0.0001 ‰ p.a. appeared with the varied two lagged variables as shown in Fig. 10c and d. They are Area I with precipitation ranging from 0 to 4 mm/day and temperature from 20 to 31.7 °C, Area II with precipitation from 7 to 10.6 mm/day and temperature from 23 to 31.7 °C, Area III with precipitation ranging from 2 to 8 mm/day and temperature from 13 to 18 °C, Area VI with precipitation near 0 mm/day and temperature near 12 °C, and Area V with precipitation from 1 to 2 mm/day and temperature near 0 °C.

**Fig. 11:**
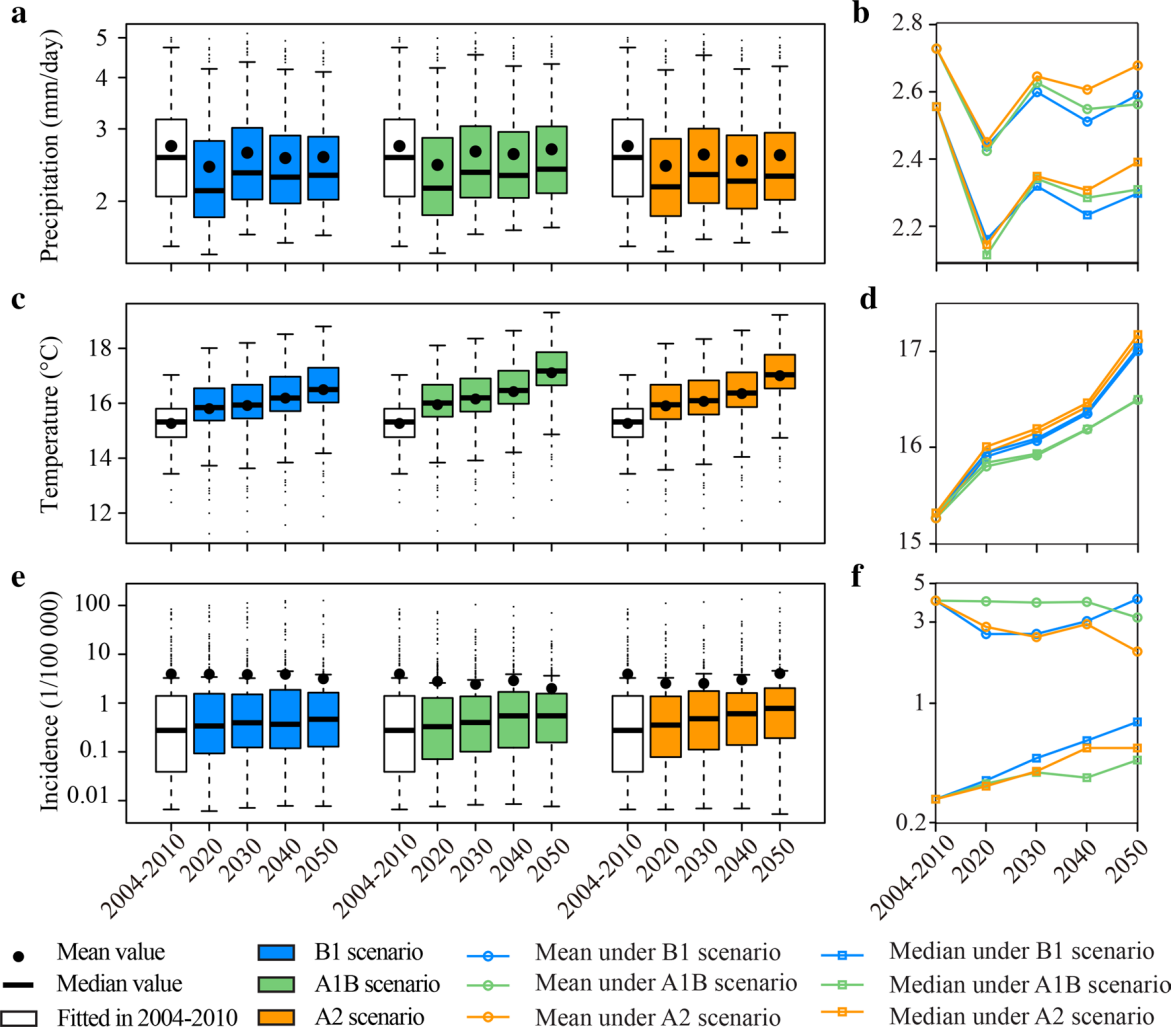
Summaries of malaria incidences under climate change scenarios with the future varied precipitation and temperature

Annual future malaria incidences derived from the sum of monthly predicted incidences using GP-based models were summarized in Fig. 11, together with the future precipitation and temperature changes. This analysis indicated that with fluctuated precipitation and the increased temperature, the median incidences would significantly increase during the studied future periods. If no actions were taken, incidence in northern China would increase 19 to 29 % in 2020, 43 % to 73 % in 2030, 33 to 119 % in 2040 and 69 to 182 % in 2050. This trend was identical with the projections of malaria vectors distribution under climate change scenarios in China [10]. But the mean incidences would not increase even decrease under SRES family B1, A1B and A2 scenarios. The integration of this result and the malaria incidences changes across the space in the future that changes primarily appeared in counties along the Huaihe River and Yangtze River shown in Fig. 9 demonstrated that the incidences in the clustering high-risk regions would decrease, but those in their surrounding regions would significantly increase and the high-risk regions would be enlarged. Under SRES family scenarios, all spatial scales of the increased incidences were enlarged in 2020, 2030, 2040 and 2050, and the decreased incidences appeared in the central high-risk areas. The comparison between these predictions and China was on the malaria elimination phase in 2014 reported in World Malaria Report 2015 [1] showed that the strategies and actions of China on malaria elimination were effective.

There are some limitations to this research. Two variables derived from remote sensing data were used for malaria prediction, but a great many remote sensing products were not explored. In previous studies as well as in this research, variables derived from remote sensing data are primarily selected based on the general theory of malaria transmission processes, and computed with correlation analysis or regression methods. The effects of various variables, however, are spatially different. Therefore, a variable explaining the malaria incidence in one location might be not appropriate or significant for other locations. In future work, more variables stemming from remote sensing data will be explored and their effects at different locations taken into consideration. In addition, the performance of averaging raster variables at county level is tested in this research, which shows that the variation of variables within most of the countries has no negative impact on the averaging process. But there are still variable variations within a few countries. Thus the uncertainties of the county-level averaging process should be further tested and addressed.

## Conclusions

Northern China is a typical mid-latitude high-risk malaria area. The combination of GP and GIS methods models well the nonlinear relationships between predictor variables and malaria incidence, to predict the future spatial distributions of malaria. The key benefit of GP is that no final solution form is assumed before constructing the relationships, unlike the forms of traditional linear regression and nonlinear model such as GAM, which are determined in advance. Thus, GP uses a proper function form instead of coefficients, as in linear regression and GAM. As a result, the GP method is able to more accurately predict malaria incidence, compared with a linear regression approach and GAM, for both training and test data. With the nonlinear relationships constructed by the GP-based prediction model, the malaria incidences in 2020, 2030, 2040 and 2050 under future climate change scenarios were predicted, mapped and analyzed. In northern China, with fluctuated precipitation and increased temperature that have one-month lagged effects on malaria incidence, the median incidence would significantly increase that it would increase 19 to 29 % in 2020, but by 2020, malaria would be eliminated in China, which indicated that the effective strategies and actions had been taken. While, the mean incidences would not increase even reduce, since the incidences in high-risk regions would reduce while the areas of high-risk regions would be enlarged.

## Abbreviations

ARE: average relative error
CCAFS: climate change, agriculture and food security
CDC: center for disease control and prevention
CI: confidence interval
CMIP3: coupled model intercomparison project phase 3
GAM: generalized additive model
GCM: global climate model
GDP: gross domestic product
GIS: geographic information system
GP: genetic programming
LAADS: level 1 and atmosphere archive and distribution system
LST: land surface temperature
MODIS: moderate resolution imaging spectroradiometer
MSSE: mean sum squared error
p.a.: per annum
PR: precipitation rate
RR: relative risk
SAD: sum of the absolute difference
SRES: specific report on emission scenarios
SRTM3: shuttle radar topography mission
TRMM: tropical rainfall measuring mission
WCRP: world climate research programme
WDI: water density index
